# Serotonin 5-HT_2A_ Receptor Function as a Contributing Factor to Both Neuropsychiatric and Cardiovascular Diseases

**DOI:** 10.1155/2009/475108

**Published:** 2009-10-13

**Authors:** Charles D. Nichols

**Affiliations:** Department of Pharmacology and Experimental Therapeutics, LSU Health Sciences Center, New Orleans, LA 70112, USA

## Abstract

There are high levels of comorbidity between neuropsychiatric and cardiovascular disorders. A key molecule central to both cognitive and cardiovascular function is the molecule serotonin. In the brain, serotonin modulates neuronal activity and is actively involved in mediating many cognitive functions and behaviors. In the periphery, serotonin is involved in vasoconstriction, inflammation, and cell growth, among other processes. It is hypothesized that one component of the serotonin system, the 5-HT_2A_ receptor, is a common and contributing factor underlying aspects of the comorbidity between neuropsychiatric and cardiovascular disorders. Within the brain this receptor participates in processes such as cognition and working memory, been implicated in effective disorders such as schizophrenia, and mediate the primary effects of hallucinogenic drugs. In the periphery, 5-HT_2A_ receptors have been linked to vasoconstriction and hypertension, and to inflammatory processes that can lead to atherosclerosis.

Neuropsychiatric disorders have high levels of comorbidity with cardiovascular disease. A recent retrospective study indicates that metabolic syndrome was reported in about 40% of schizophrenic patients, 35% of bipolar patients, and 25% of patients with recurrent depression [1]. Environmental factors, including medications, likely underlie some of the metabolic dysfunction associated with schizophrenia and depression, however, studies in unmedicated drug naïve first episode schizophrenics indicate that a pathological association exists [2]. Significantly, many other studies have also linked metabolic syndrome, cardiovascular disease, and psychiatric disorders [3–7], and specific aspects of cardiovascular disease like atherosclerosis and hypertension are associated with psychiatric disorders [8–10]. Patients with schizophrenia have an average reduction in life expectancy of 15 years, largely due to coronary heart disease [11]. Unfortunately, many therapeutics used to treat psychiatric disorders can have significant negative influences on aspects of cardiovascular function and have thus clouded the nature of these links with regard to cause and effect. Antipsychotic medications, as well as therapeutics for other psychiatric disorders, can have dramatic effects on metabolic processes and can induce metabolic syndrome, weight gain, and diabetes, which are all significant risk factors for the development of cardiovascular diseases [12–15]. Furthermore, prolongation of the interval between ventricular depolarization and repolarization (QT interval) also has been associated with antipsychotic medications [16]. Overall, metabolic and cardiovascular dysfunction associated with neuropsychiatric disorders, therefore, likely represent a mixture of environmental, medication, and pathological factors.

Whereas the exact biochemical nature of the links between cardiovascular disease and psychiatric disorders remains elusive, it is evident that there is a strong association between these biological processes. The fact that medications used to treat one condition can influence, and even induce, the other condition underscores these associations. With respect to depression, models have been proposed that largely invoke an underlying dysregulation of the HPA axis, which through modulation of factors such as cortisol and CRF influence mood, affect, immunity, and cardiovascular function [6, 17, 18].

Aspects of cardiovascular disease including endothelial dysfunction and atherosclerosis are acutely mediated by inflammatory mechanisms. For example, adipose tissues can release proinflammatory cytokines into the circulation. As more adipose tissues are present in an individual, represented by a higher body mass index, more cytokines can be released. These cytokines, primarily Tumor Necrosis Factor-*α* (TNF-*α*) and IL6, can directly induce inflammation in cardiovascular tissues, as well as activate the HPA axis, which in turn can lead to metabolic syndrome. Metabolic syndrome can subsequently lead to oxidative stress and generation of free radicals that together induce further production of proinflammatory cytokines, and the two processes of inflammation and metabolic syndrome can interact synergistically to elevate levels of proinflammatory cytokines and promote further endothelial dysfunction and atherosclerosis [19]. A detailed review of the development and progression of atherosclerosis itself will not be given here, and the reader is referred to other reviews and references therein [20, 21]. A key mediator of the development of atherosclerosis is the cytokine TNF-*α* which, acting through its receptors on the surface of macrophage, endothelial, and smooth muscle cells of the vasculature, induces signal transduction cascades leading to NOS activity, activation of transcription factors such as Nuclear Factor kappa B (NF-*κ*B), and production of proinflammatory adhesion molecules and cytokines such as ICAM-1, VCAM-1, and IL6. Together, these processes facilitate macrophage infiltration of the arterial wall, differentiation of macrophages to lipid-accumulating foam cells, and migration of arterial smooth muscle cells to form a fibrous cap, together constituting the atherosclerotic plaque. Severe cases cause significant blockage of the artery, and eventual rupture of the plaque and thrombosis. 

Recently, cytokine-mediated inflammation has been implicated in the development and presentation of psychiatric disorders that include depression and psychosis [22–24]. In major depression and bipolar disease, increases in TNF-*α*, and other proinflammatory cytokines (e.g., IL6, and other proinflammatory molecules such as ICAM-1 and MCP-1), have been found within the CNS [23, 25]. Although the association of inflammation with depression does not necessarily imply causality, certain symptoms of depression have been shown in both clinical studies and animal models to be alleviated by anti-inflammatory therapeutics [26]. Interestingly, knockout mice lacking TNF-*α* receptors exhibit antidepressant-like behaviors in several types of assays [27]. Neuroinflammation leading to dysfunction of the adult CNS as well as inflammatory events in utero leading to perturbation of normal synaptic development has been proposed as possible factors contributing to psychiatric disorders [23, 24]. 

It has long been recognized that 5-hydroxytryptamine (serotonin; 5-HT), and its biosynthetic precursor tryptophan, play an important role in regulating immune functions through non-5-HT receptor interactions involving circulating tryptophan and kynurenine levels [28–30]. Individual serotonin receptors, however, are expressed in many immune-related tissues, and interactions at specific receptors are also known to modulate aspects of the immune response and inflammation [31–33]. Within the CNS, serotonin and serotonin receptors have been strongly associated with normal function. Certain neuropsychiatric disorders that include depression, bipolar disorder, OCD, anorexia, and schizophrenia have been linked to dysregulation of CNS serotonin [34, 35]. Indeed, therapeutics for these disorders often include inhibition of the serotonin transporter (SERT) with selective serotonin reuptake inhibitor (SSRI) medications, or blockade of specific serotonin receptor subtypes. SSRIs can also show an efficacy in treating aspects of cardiovascular disease associated with depression [36], and have been demonstrated in animal models to have an anti-inflammatory effect [37]. The mechanisms underlying the protective effect of antidepressants are not precisely known, but are predicted by some researchers to involve activation of the pituitary-adrenocortical system via increased central serotonin levels [38], by modulation of cytokine levels in peripheral tissues [39, 40], and by suppression of platelet activation [41]. Furthermore, acute SSRI administration has been shown to have a vasodilatory effect on the coronary artery that may be cardioprotective [42]. Interestingly, TNF-*α*, as well as certain other cytokines, have been shown to influence both expression and transport activity of the serotonin transporter. In neuronally derived cells and choriocarcinoma cells, TNF-*α*, INF-*γ*, and IL1*β* increase function [43–45], whereas in B lymphocytes, IL4 decreases function [46], and in intestinal epithelial derived Caco-2 cells, TNF-*α* has been found to decrease both expression and transport activity of SERT [47]. Whereas the nature of the influence of cytokines on SERT function (e.g., facilitation or repression) likely depends on the cytokine and tissue, modulation of synaptic serotonin levels in various brain regions by inflammatory cytokines would certainly be anticipated to have some effect on neuronal function relevant to psychiatric disorders like depression. In summary, there appears to be a strong link between proper functioning and regulation of the serotonin system and factors underlying cardiovascular disease and neuropsychiatric disorders.

We hypothesize that a particular aspect of the serotonin system, the 5-HT_2A_ receptor, is a common and contributing factor underlying aspects of normal cardiovascular and CNS function, and that dysfunction of this receptor results in certain characteristics of cardiovascular and neuropsychiatric disorders. There are seven families of serotonin receptors comprised of fourteen distinct subtypes [48]. With the exception of the 5-HT_3_ receptor, which is a ligand-gated ion channel, all are seven transmembrane-spanning G-protein-coupled receptors. Of all the serotonin receptors, the 5-HT_2A_ receptor has been the one most closely linked to complex behaviors and neuropsychiatric disorders. The 5-HT_2A_ receptor is highly expressed within the frontal cortex, with lower expression levels throughout the brain [48]. There has been extensive research performed to establish the role of 5-HT_2A_ receptors within the brain, where they have been shown to participate in processes such as cognition and working memory [49], mediate the primary effects of hallucinogenic drugs [50], and been implicated in mechanisms underlying schizophrenia [51, 52]. Furthermore, abnormal expression of 5-HT_2A_ receptors has also been linked to depression. For example, some studies have shown that receptor protein expression is increased in certain cortical areas of patients with major depression [53, 54], as well as suicide victims [55, 56]. 5-HT_2A_ receptor expression decreases, however, have been found in brain limbic regions of patients with major depressive disorder [57].

Significantly, 5-HT_2A_ receptors are found outside the CNS in many diverse tissues, including those related to cardiovascular function. Their role in the periphery, however, is less clear. Also, 5-HT_2A_ receptor mRNA is expressed within vascular smooth muscle and endothelial cells, and cardiomyocytes, where the receptors are believed to mediate aspects of vasoconstriction and cellular proliferation [58–60]. Not only can 5-HT_2A_ receptor activity modulate cardiovascular function in the periphery, but it has been found to act centrally: activation of 5-HT_2A_ receptors in the nucleus tractus solitarius of the brain dramatically lowers both blood pressure and heart rate [61].

Recently, we have found that selective activation of 5-HT_2A_ receptors in primary aortic smooth muscle inhibits TNF-*α*-mediated inflammatory markers with extraordinary potency. With an IC_50_ value of about 10 picomolar, 5-HT_2A_ receptor activation with the drug (*R*)-DOI inhibits NOS activity, the activation and nuclear translocation of the p65 subunit of NF-*κ*B, as well as the production of mRNA for the proinflammatory cell adhesion proteins ICAM-1 and VCAM-1, and mRNA for the cytokine IL6 [33]. Other chemically diverse molecules that activate 5-HT_2A_ receptors, including the hallucinogen lysergic acid diethylamide (LSD), also have potent anti-inflammatory effects on aortic smooth muscle in vitro [33], indicating that this is a property of 5-HT_2A_ receptor activation and not specific to a particular drug. Significantly, we have found potent anti-inflammatory effects in primary aortic endothelial cells as well as macrophages (unpublished data). TNF-*α* signaling in these three cell types, aortic smooth muscle, endothelial, and macrophage, is believed to be a major contributing factor to the inflammatory processes underlying the development and progression of atherosclerosis. As such, drugs acting at 5-HT_2A_ receptors, like (*R*)-DOI, may represent a novel class of superpotent small molecule inhibitors of TNF-*α* pathway signaling with therapeutic potential for treating not only atherosclerosis but also other inflammatory conditions involving TNF-*α ,* that are more then 100-fold more potent than the more potent steroidal anti-inflammatories currently on the market. Importantly, we have also found potent anti-inflammatory effects of 5-HT_2A_ receptor activation in CNS-related cell culture systems, including C6 glioma, and SH-SY5Y neuroblastoma cells (unpublished data), indicating that the role of 5-HT_2A_ receptors in mediating anti-inflammatory pathways is not limited to cardiovascular tissues, but is likely relevant in the CNS.

As mentioned previously, drugs that interact with or influence 5-HT_2A_ receptor function can dramatically affect aspects of cardiovascular function. Some, including atypical antipsychotic, medications have a negative influence, while others, including ketanserin and certain antidepressants, are reported to have a beneficial cardiovascular effect. How do these effects fit within the framework of our hypothesis?

Ketanserin has been effective in the clinic as an antihypertensive agent as well as an antiarrhythmic. It can also sometimes induce proarrhythmias, and was withdrawn from the market largely for this reason. Recent reports suggest that the antiarrhythmic effects of ketanserin may be due to direct interactions with certain potassium channels, including the HERG channel, and not to blockade of the 5-HT_2A_ receptor per se [62–64]. With regards to ketanserin's use as an antihypertensive, the underlying mechanisms are not entirely clear as ketanserin has significant affinity for the alpha-1 adrenergic receptor, and many reports have cited this as the putative antihypertensive therapeutic target rather than antagonism of the 5-HT_2A_ receptor [58, 65, 66]. Nevertheless, many in vitro studies of 5-HT_2A_ receptor antagonists have clearly demonstrated that 5-HT-induced vasoconstriction in isolated vascular tissue preparations is in large part mediated by 5-HT_2A_ receptors [60]. Although blockade of 5-HT_2A_ receptors can potently inhibit serotonin-mediated vasoconstriction in isolated vascular preparations, aside from ketanserin, other 5-HT_2A_ receptor antagonists show little to no antihypertensive effect in vivo [66, 67]. Indeed, newer highly selective 5-HT_2A_ receptor antagonists, like M100907 (volinanserin), ACP-103 (primavanserin), and SR46349B (eplivanserin), are currently in clinical trials as novel therapeutics to treat insomnia [68] and there are no reports in literature describing effects on hypertension, inflammation, or other cardiovascular processes. One report, however, examining the physiological and pharmacokinetics of ACP-103 in a small study comprised of normal human subjects has been published that concluded that there were no significant changes in vital signs or ECG associated with treatment for up to fourteen days [69].

An interesting study recently published detailed the effects of chronic increases in circulating serotonin levels, as opposed to large bolus doses. It was predicted that, as occurs with a bolus dose of serotonin, blood pressure would increase due to the vasoconstrictive effects of increased 5-HT acting at 5-HT_2_ receptors. It was found that increased circulating 5-HT levels actually significantly *decreased* blood pressure [70, 71]. The author of this study stated that it was unlikely that direct activation of vasoralaxant 5-HT receptors was responsible for this effect, and that further studies are needed to elucidate underlying mechanisms [70]. If antagonism of 5-HT_2A_ receptors is expected to produce hypotension and affect cardiac rhythmicity, then activation would be anticipated to produce hypertension and potentially affect rhythmicity. This has not been the case. In humans, the 5-HT_2A_ receptor agonist, psilocybin, which also has high affinity for 5-HT_1A_ receptors, produces only mild and transient cardiovascular effects at high doses when administered systemically. Highly hallucinogenic doses (e.g., 30 mg) only produce minor and transient increases in baseline heart rate (+10 bpm) and blood pressure (~15%) and do not influence heart function as measured by electrocardiogram [72–74]. Lower non-hallucinogenic doses of psilocybin do not produce significant changes in heart rate, blood pressure, or heart function [72–74]. Another 5-HT_2A_ receptor agonist dimethyltryptamne (DMT) has been given to humans at highly hallucinogenic doses [75]. In that study, intravenous injection of DMT was found to only elicit minor and very transient increases in heart rate and blood pressure [75]. It should be noted that some of these increase can probably be attributed to psychological stress and anxiety produced by the hallucinogenic effects of psilocybin and DMT at high doses, and not by a direct pharmacological action on blood pressure or heart rate. There have been no studies reported examining the effects of chronic administration of 5-HT_2A_ receptor agonists in mammals. It will be interesting to see in future experiments if chronic administration of these agents affects inflammation-related cardiovascular diseases or other aspects of cardiovascular function. Our data indicate that potential anti-inflammatory effects of agonists like (*R*)-DOI would be evident at doses far below that necessary to elicit behavioral effects like hallucinations. Interestingly, there are antidepressant-like effects associated with single hallucinogenic doses of psilocybin [73, 76].

Atypical antipsychotic medications like olanzapine, clozapine, and risperidone belong to a newer class of drug that are believed to have a component of their therapeutic effect mediated by antagonism of 5-HT_2A_ receptors [77]. Unlike traditional antipsychotic medications like haloperidol that act primarily as antagonists at dopamine D2 receptors, atypical antipsychotics have some efficacy at treating the negative, or more cognitive, symptoms of schizophrenia, and this may be due to their effects on 5-HT_2A_ receptors. As previously mentioned, pathological associations exist between schizophrenia and metabolic syndrome and cardiovascular disorders, however, the use of atypical antipsychotics is, unfortunately, strongly associated with the development of significant weight gain, metabolic, and cardiovascular disorders [14, 15, 78]. The substantial weight gain associated with atypical antipsychotics is believed to partially involve antagonist or inverse agonist activity of these drugs at 5-HT_2C_ receptors [79]. Indeed, the 5-HT_2C_ knockout mouse is severely obese [80], and agonists of this receptor can produce hypophagia [81]. Although many aspects of metabolic and cardiovascular disorders associated with atypical antipsychotics are likely a direct consequence of weight gain, other aspects may be mediated by blockade of 5-HT_2A_ receptor function. For example, 5-HT_2A_ receptors have been implicated in regulation of glucose homeostasis [82, 83], and antagonism of the 5-HT_2A_ receptor may influence insulin sensitivity [84, 85]. Within the framework of our hypothesis, aberrant 5-HT_2A_ receptor function may contribute to both psychosis and pathological association of metabolic and cardiovascular disorders. This dysfunction could result in hyperacticvity in the CNS, and contribute to psychosis. In the periphery, receptor dysfunction may promote processes leading to metabolic disorder and cardiovascular disease through largely unexplored mechanisms. Whereas blockade of 5-HT_2A_ receptor hyperfunction in the CNS may be therapeutic for treating psychosis, receptor blockade, both in the CNS and periphery, may also interfere with endogenous anti-inflammatory processes and synergistically act with the effects of induced weight gain to produce significant metabolic and cardiovascular disorders. 

Another class of medication that affects psychiatric disorders, inflammatory processes, and cardiovascular function is selective serotonin reuptake inhibitor antidepressants (SSRIs). Interestingly, SSRI antidepressant medications have a biphasic effect on serotonin within the brain. Acute treatment leads to decreased serotonin release, and chronic treatment leads to increased release [86, 87]. The acute decrease in 5-HT release results from autoreceptor activation and subsequent inhibition of release and synthesis of serotonin. As these receptors desensitize with chronic SSRI treatment, however, overall 5-HT transmission is facilitated. Chronic treatment with SSRI antidepressants also has been shown to produce significant downregulation and desensitization of 5-HT_2A_ receptors both in vitro and in vivo similar to chronic treatment with atypical antipsychotics [88]. The effects of SSRI induced receptor desensitization and downregulation would be anticipated to mimic the effects of chronic treatment with atypical antipsychotics, and reduce overall 5-HT_2A_ receptor function. Within the framework of our model, these effects would be predicted to produce a deficit in receptor function, and increases in proinflamatory mechanisms potentially leading to cardiovascular disease, metabolic disorders, and neuroinflammation. SSRI antidepressants, however, have been shown to have anti-inflammatory activity and to be cardioprotective when given both acutely and chronically. It is conceivable that the acute anti-inflammatory and cardioprotective effects of SSRI antidepressants are mediated by mechanisms other than manipulation of 5-HT_2A_ receptor function, as discussed previously, and the beneficial effects of chronic treatment may involve enhanced 5-HT tone at 5-HT_2A_ receptors. Although chronic treatment with SSRI antidepressants produces desensitization and downregulation of 5-HT_2A_ receptors, our results demonstrate that 5-HT_2A_ receptors in this state are actually *more sensitive* to the anti-inflammatory effects of activation by the agonist (*R*)-DOI by an order of magnitude [33]. Together, the anti-inflammatory and cardioprotective effects of SSRI antidepressants are, therefore, likely a combination of direct modulation of cytokines, central action within the CNS, and modulation of 5-HT_2A_ receptor function, with each component contributing differently as therapy progresses to achieve a steady state.

Here, we propose that deficits in 5-HT_2A_ receptor function underlie at least part of the comorbidity of cardiovascular disease and neuropsychiatric disorders. If 5-HT_2A_ receptor activation normally appears to exert a powerful anti-inflammatory influence on a variety of cells, especially vascular tissues, dysfunction may be anticipated to lead to a repression of anti-inflammatory influences and to the expression of proinflammatory markers, sensitization of the cell to inflammatory stimuli, or both, leading to an increased risk of inflammation and atherosclerosis. Similarly, 5-HT_2A_ receptor dysfunction also may contribute to increased risk of hypertension, and cardiac hypertrophies within the cardiovascular system. Unfortunately, there are few, if any, studies reported in literature examining expression levels of 5-HT_2A_ receptors in diseased cardiovascular related tissues. This simply may be due to the fact that no one has looked. If so, then examination of receptor levels in diseased cardiovascular-related tissues may be a productive avenue of exploration. In rat models of congestive heart failure, there are two reports demonstrating increased levels of 5-HT_2A_ receptor mRNA [59, 89]. It remains to be determined if the increased expression is causative, or a compensatory response to other factors.

Within the CNS, the same receptor dysfunction may result in or contribute to the development of neuropsychiatric disorders including depression, bipolar disease, and psychosis. This dysfunction may either come from alterations in regulation due to promoter polymorphisms or other regulatory mechanisms influencing expression, or polymorphisms or mutations affecting the protein itself that could influence responsiveness and downstream signal transduction pathways. Polymorphisms in the promoter region of the human HTR2A locus have been shown to alter receptor expression levels [90], and these same polymorphisms have been linked to response to antisychotics and certain SSRIs [91, 92], and in some studies positively associated with various CNS conditions including major depression, bipolar disorder, and schizophrenia [93–96]. Significantly, positive associations also have been detected for these polymorphisms and symptoms of cardiovascular-related disorders [97]. Polymorphisms within the coding regions of the HTR2A locus have been found in some studies to be positively associated with neuropsychiatric disorders, as well as to rheumatoid arthritis [98], circulating cholesterol levels [99], hypertension [100], myocardial infarction [101], as well as blood pressure and metabolic syndrome [102].

There is significant opportunity for future research to investigate how 5-HT_2A_ receptor function mediates certain aspects of both neuropsychiatric and cardiovascular-related disorders. Greater clarification of the role of receptor antagonists in vivo is needed. This could involve examining the effects of the new highly selective receptor antagonists in rodent models of cardiovascular disease and atherosclerosis, as well as careful examination of clinical trial data for the use of these drugs as sleep aids and continued analysis for the effects of chronic use on cardiovascular-related issues after these therapeutics come to market. Not only could results from these types of studies be informative about the effects of selective receptor blockade on cardiovascular-related diseases but they could also help to address the question of whether or not the negative cardiovascular and metabolic effects of atypical antipsychotics have a significant 5-HT_2A_ receptor-mediated component. If they did, then perhaps long-term therapy with these new highly selective receptor antagonists would produce metabolic and cardiovascular disorders. In our laboratory, we are continuing to study the effects of agonists on inflammation-related cardiovascular processes, and attempting to elucidate the molecular mechanisms underlying their anti-inflammatory effects. An additional resource that would beneficial to explore is the 5-HT_2A_ receptor knockout mouse model. Amazingly, given the widespread expression and importance of the 5-HT_2A_ receptor, the knockout animal appears overtly normal. There are, however, certain behavioral effects associated with loss of this receptor [103, 104]. Interestingly, some observed behaviors are opposite to the effects of receptor antagonists [105], indicating that caution should be exercised in the interpretation of knockout studies using this model. Nevertheless, studies utilizing this mouse in models of cardiovascular-related diseases will likely be of value. A better understanding of the relationship between 5-HT_2A_ receptor function and its roles in both the CNS and cardiovascular system should lead to development of improved therapeutics to treat diseases affecting each of these systems either separately or together.
